# Carbon Dioxide Emissions from Reservoirs in the Lower Jordan Watershed

**DOI:** 10.1371/journal.pone.0143381

**Published:** 2015-11-20

**Authors:** Zeyad Alshboul, Andreas Lorke

**Affiliations:** University of Koblenz-Landau, Institute for Environmental Sciences, Fortstr.7, 76829 Landau, Germany; The Ohio State University, UNITED STATES

## Abstract

We have analyzed monthly hydrological, meteorological and water quality data from three irrigation and drinking water reservoirs in the lower Jordan River basin and estimated the atmospheric emission rates of CO_2_. The data were collected between 2006 and 2013 and show that the reservoirs, which differ in size and age, were net sources of CO_2_. The estimated surface fluxes were comparable in magnitude to those reported for hydroelectric reservoirs in the tropical and sub-tropical zones. Highest emission rates were observed for a newly established reservoir, which was initially filled during the sampling period. In the two older reservoirs, CO_2_ partial pressures and fluxes were significantly decreasing during the observation period, which could be related to simultaneously occurring temporal trends in water residence time and chemical composition of the water. The results indicate a strong influence of water and reservoir management (e.g. water consumption) on CO_2_ emission rates, which is affected by the increasing anthropogenic pressure on the limited water resources in the study area. The low wind speed and relatively high pH favored chemical enhancement of the CO_2_ gas exchange at the reservoir surfaces, which caused on average a four-fold enhancement of the fluxes. A sensitivity analysis indicates that the uncertainty of the estimated fluxes is, besides pH, mainly affected by the poorly resolved wind speed and resulting uncertainty of the chemical enhancement factor.

## Introduction

Inland waters represent an important component of terrestrial landscapes, playing an ecological and biogeochemical role that is largely disproportional to their areal extent [1, 2]. Only recently it has been recognized that the amount of terrestrial carbon, which is processed and eventually emitted into the atmosphere as CO_2_ from inland waters is similar in magnitude than current estimates of global net terrestrial ecosystem production [2–4]. Quantifying the role of freshwater systems in terms of carbon sinks and sources is fundamental for improving the balance approach of regional and global carbon budgets. The role of inland waters for global and regional carbon cycling is strongly affected by human activities [5, 6]. On the basis of the limited available data, it was suggested that man-made reservoirs, as a rather small part of the inland water systems, are potentially an important source of greenhouse gases to the atmosphere, with CO_2_ emission rates exceeding those of natural lakes [7–9]. The current estimates of CO_2_ emissions from inland waters are either based on syntheses and spatial upscaling of (i) few direct CO_2_ partial pressure measurements and direct flux measurements, e.g., obtained using head space technique and floating chambers [7, 9], or (ii) estimates of CO_2_ partial pressures and CO_2_ fluxes calculated from pH, alkalinity, temperature and wind speed data that are available from water quality monitoring programs and climatological stations [4, 10, 11].

Most studies on which current knowledge on reservoir greenhouse gas emissions is based on, are from regions where surface water is rather abundant, e.g. from the boreal and tropical zones [7, 8]. Representative flux measurements from reservoirs in arid and semi-arid regions, where the anthropogenic pressure on surface waters can be expected to be highest due to extensive water usage, are limited (but see [12, 13]). The lower Jordan River basin, located between Lake Tiberias and the Dead Sea, and its tributaries can be considered as an example for such systems. Surface waters in this region are expected to be highly vulnerable to climatic change [14]. About 83% of the population of Jordan and the majority of the country’s irrigated agriculture and water resources are located within the lower Jordan River basin [15]. The scarce water resources in Jordan are subject to salinization [16], which can result in chemical enhancement of water-atmosphere CO_2_ fluxes [10]. Further, high loading with organic carbon from treated and untreated waste water [17] and high sediment yield from intense agricultural land use [18] provide favorable conditions for aerobic and anaerobic C-degradation and comparably high atmospheric emission rates of CO_2_.

The objective of this study is to estimate the CO_2_ fluxes from irrigation and drinking water reservoirs in the lower Jordan River basin. We use water quality and meteorological data from three reservoirs for the time period 2006 to 2013 to estimate the CO_2_ partial pressures and the wind-speed dependent gas exchange velocities. The resulting fluxes were analyzed statistically to identify potential temporal trends and correlations to available hydrological data. We relate our findings to the current estimates of CO_2_ emissions rates from hydropower reservoirs and natural lakes in different climatic zones and discuss potential regional-specific drivers for flux variations.

## Materials and Methods

### 2.1 Study sites

**Fig 1:**
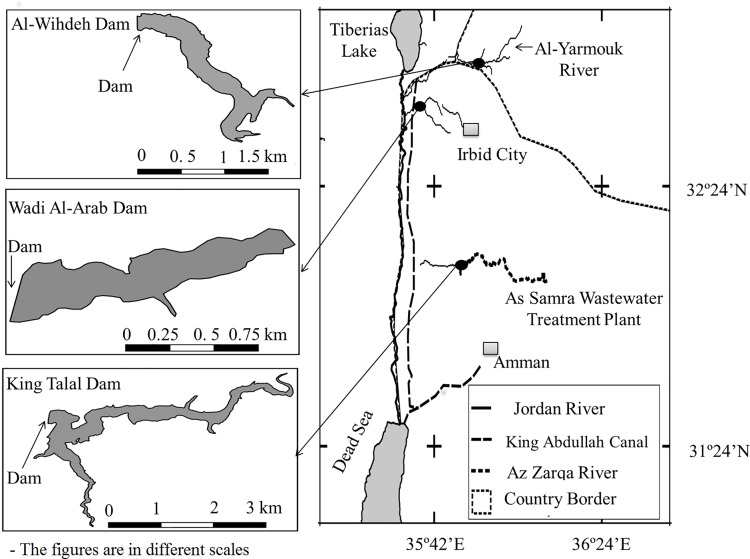
The map on the right shows the location of the three reservoirs (King Talal Dam, Al-Wihdeh Dam and Wadi Al-Arab Dam) within the lower Jordan watershed between Lake Tiberias and the Dead Sea. The morphological characteristics of the reservoirs are shown in the detailed maps on the left at different scales.

We analyzed data from three main reservoirs located in the northern part of the lower Jordan watershed: King Talal Dam, Al-Wihdeh Dam and Wadi Al-Arab Dam (Fig 1). The reservoirs differ in surface area, catchment size, water quality, and outflow rate. The main characteristics of these reservoirs are summarized in Table 1 and briefly described below.

**Table 1:** The main physical characteristics of the three reservoirs. Numbers are provided as mean±SD (standard deviation), if available.

	Wadi Al-Arab Dam	King Talal Dam	Al-Wihdeh Dam
Surface area (km^2^)	0.56±0.1	1.6±0.26	0.82±0.44
Total capacity (10^6^ m^3^)	16.9	74	110
Dam height (m)	83.5	108	87
Main tributaries	- King Abdullah Canal	- Az Zarqa River	- Al-Yarmouk River
	- Al-Yarmouk River	- Effluent of As-Samra WWTP	
Outflow (10^6^ m^3^ y^-1^)	11.4**±**1.8	98.5**±**8.9	14.9**±**4.5
Year of construction	1987	1986	2006
Usage	Agriculture, industrial and domestic	Agriculture	Agriculture and domestic

King Talal Dam is the largest reservoir in the northern part of Jordan and it is designed to supply water for irrigation in the Jordan valley [17]. The reservoir receives its inflow from the surrounding tributaries and from the As Samra Wastewater treatment plant. The tributaries include the winter floods and the effluent of the Az Zarga River desalination plant, which is governed by the Jordanian Ministry of Water and Irrigation. The desalination plant is fed by the surrounding ground waters and started to operate in 2008. As Samra Wastewater treatment plant was constructed in 1985 and was expanded between 2006 and 2010 to treat 79% of all wastewater generated in Jordan [19]. Its effluents are discharged in the Az Zarqa River, which receives its water from the springs of Amman City.

Al-Wihdeh Dam is located in the trans-boundary basin of the Al-Yarmouk River, between Jordan and Syria. The dam began to receive water in 2006, while the construction was completed in 2009. Al-Wihdeh Dam is mainly fed by the winter floods and available base flow from the upstream located catchment of the Al-Yarmouk River including groundwater [20].

Wadi Al-Arab Dam is located in the northern part of Jordan, 10 km south of Lake Tiberias. The reservoir mainly receives waters from the King Abdullah Canal and surrounding tributaries [21]. The reservoir waters are used for irrigation and during periods of water shortage also for human consumption. More information on water quality and physico-chemical features of Wadi Al-Arab Dam can be found elsewhere [22].

### 2.2 Data

Water temperature, pH, alkalinity, specific conductance and the concentrations of calcium, magnesium, potassium, sodium and chloride, were obtained for all three reservoirs from monthly measurements conducted by the Water Authority of Jordan (S1 Dataset. Physico-chemical and hydrological parameters of the three studied reservoirs.). The water samples were collected below the surface at the outflow of the reservoirs. pH, specific conductivity and water temperature measurements were conducted using a portable pH/conductivity/temperature meter (Hanna, Hannainst, USA: pH: 0–14 ±0.05; conductivity: 0–3999 μScm^-1^ ±1 μScm^-1^; temperature: 0–60°C ± 0.1°C). The meter was weekly calibrated following the instructions of the manufacturer using standard buffers for pH (4.01, 7.01 and 10.01) and conductivity (1413 μScm^-1^). Water samples were titrated for alkalinity measurements with sulfuric acid to an equivalent point of pH 4.5 (±0.018 mmol l^-1^). The major ions were analyzed with ion chromatograph (Dionex, USA), equipped with an analytical column (CS12, 4×250mm), a guard column (CS12, 4×50mm) and a self-regenerating suppressor (CSRS- ΙΙ,mm) for cations, and an analytical column (Ionpac® anion AS4A-SC, 4×250mm), a guard column (AG4A-SC, 4×50mm) and a self-generating suppressor (ASRS-Ι, 4mm) for ions. The water chemistry data of King Talal Dam and Al-Wihdeh Dam are available from August 2006 to April 2013 and the data for Wadi Al-Arab Dam are available from April 2007 to April 2013.

Hourly values of wind speed at 10 meter height (*U*_10_) at King Talal Dam and Al-Wihdeh Dam were obtained from the metrological stations located at the dams and operated by the Jordan Valley Authority. The *U*_10_ data for Wadi Al-Arab Dam were obtained from the National Oceanic and Atmospheric Administration (NOAA, http://www.noaa.gov/index.html) as the mean value of three hours measured at Irbid and Ghor Safi climatological stations. The mean *U*_10_ value between the water sampling dates was used in our calculations.

The daily mean of the outflow, inflow, water level, water volume and the water surface area as a function of water levels, for all three reservoirs were obtained from the database of Jordan Valley Authority (JVA, http://www.jva.gov.jo/sites/en-us/default.aspx). (See also supporting information, S1 Dataset. Physico-chemical and hydrological parameters of the three studied reservoirs.)

### 2.3 Calculations and analyses

The concentration of dissolved gaseous CO_2_ (mol l^-1^) was calculated from the available data for alkalinity, pH, water temperature and ion concentrations by assuming chemical equilibrium conditions using the software PHREEQC v3 [23]. The concentration of CO_2_ was converted to partial pressure *p*CO_2_ using the temperature dependent Henry constant *K*_*H*_(mol l^-1^ atm^-1^) provided by *Wanninkhof* [1992].

The atmospheric flux of CO_2_ (mol m^-2^ d^-1^) at the reservoirs surface was estimated following Wanninkhof and Knox [24]

$$Flux=k\prime_{CO_{2}}\;\alpha \;K_{H}(p{\text{CO}}_{2}{}_{,aq}\;-\;p{\text{CO}}_{2}{}_{,\text{air}})$$ (1)

where $k\prime_{{}_{{\text{CO}}_{2}}}$ is the gas exchange velocity (m d^-1^) for CO_2_, *α* describes the chemical enhancement of the CO_2_ flux, *K*_H_ is the Henry constant (mol l^-1^ atm^-1^), *p*CO_2,aq_ and *p*CO_2,air_ (μatm) are the partial pressures of CO_2_ in water and the atmosphere, respectively. The atmospheric partial pressure *p*CO_2,air_ was considered to be constant (396 μatm), corresponding to the global mean value in March 2013 (http://esrl.noaa.gov).

The gas exchange velocity $k\prime_{{}_{{\text{CO}}_{2}}}$ was estimated from the wind-speed dependent exchange velocity at a Schmidt number of 600 (*k’*_600_) and the temperature-dependent Schmidt number of CO_2_ (*Sc*_600_) [25]

$$k\prime_{CO_{2}}=k\prime_{600}{\left(\frac{600}{Sc_{600}}\right)}^{n}$$ (2)

We used a Schmidt number exponent *n* of 2/3 and 1/2 for *U*_10_ below and above 3.7 m s^-1^, respectively [26]. *k*′_600_ was calculated as a function of wind speed using the bilinear steady wind relationship according to Crusius and Wanninkhof [25].

$$k\prime_{600}({\text{cm h}}^{-1})=\left\{\begin{array}{l} 0.72U_{10}\;,\;U_{10}<3.7\;{\text{m s}}^{-1}\\ 4.33U_{10}\;-\;13.3\;,\;U_{10}\geq 3.7\;{\text{m s}}^{-1} \end{array}\right\}$$ (3)

The exchange of CO_2_ at the water surface can be enhanced in comparison to other gases by the dissociation of carbonic acid within the diffusive boundary layer [24]. The hydration reaction rates for CO_2_ are functions of temperature, pH and ion concentrations. If the reaction time scales are the same order of magnitude as the residence time of the CO_2_ molecules in the aqueous boundary layer, both chemical reaction and diffusion are important for CO_2_ exchange at the air-water interface [24]. The chemical enhancement factor *α* was calculated using the model of Hoover and Berkshire [27]. The calculations include the first and second apparent dissociation constants for carbonic acid according to Dickson and Millero [28] and the hydration rate constant of carbonic acid and bicarbonate according to Johnson [29]. The model further includes water salinity, which was calculated from specific conductance using the empirical approximation provided by Duarte *et*. *al*. [10].

The annual mean retention time (d) for each reservoir was estimated by dividing the annual mean water volume (m^3^) by the mean outflow rate (m^3^ d^-1^). The daily mean of the water surface area was calculated from the daily mean water level, using linear regressions of the measured water surface area and water level for the three reservoirs at different dates. The emission rate of CO_2_ was multiplied by the daily mean surface area of the reservoirs to estimate the total mean emission rates from the three reservoirs (g CO_2_ yr^-1^).

The relative importance of the variations of the input variables alkalinity, pH, temperature and salinity for the estimated variations of *p*CO_2_ and CO_2_ flux was evaluated by performing additional calculations using the constant mean values of the respective variables. The same analysis was conducted for evaluating the effect of dissolved ions by comparing the estimates of *p*CO_2_ and flux with estimates for which ions were omitted in the chemical equilibrium calculations. The relative importance of variations of wind speed for the estimated variations of the CO_2_ flux was evaluated by using a constant mean wind speed instead of measured time series.

Most of the parameters involved in our calculations violate normality assumptions. Thus, the nonparametric Spearman’s rank correlation coefficient (*ρ*) was used and the correlations among measured and estimated variables were considered at the significance level of *p*<0.05, unless stated otherwise. Temporal trends were estimated by linear regression at a significance level of *p*<0.05. The temporal trends of the variables were analyzed for years with complete data coverage, i.e. between 2007 and 2012 for King Talal Dam and Al-Wihdeh Dam, and between 2008 and 2012 for Wadi Al-Arab Dam.

## Results

### 3.1 Physico-chemical characteristics

**Fig 2:**
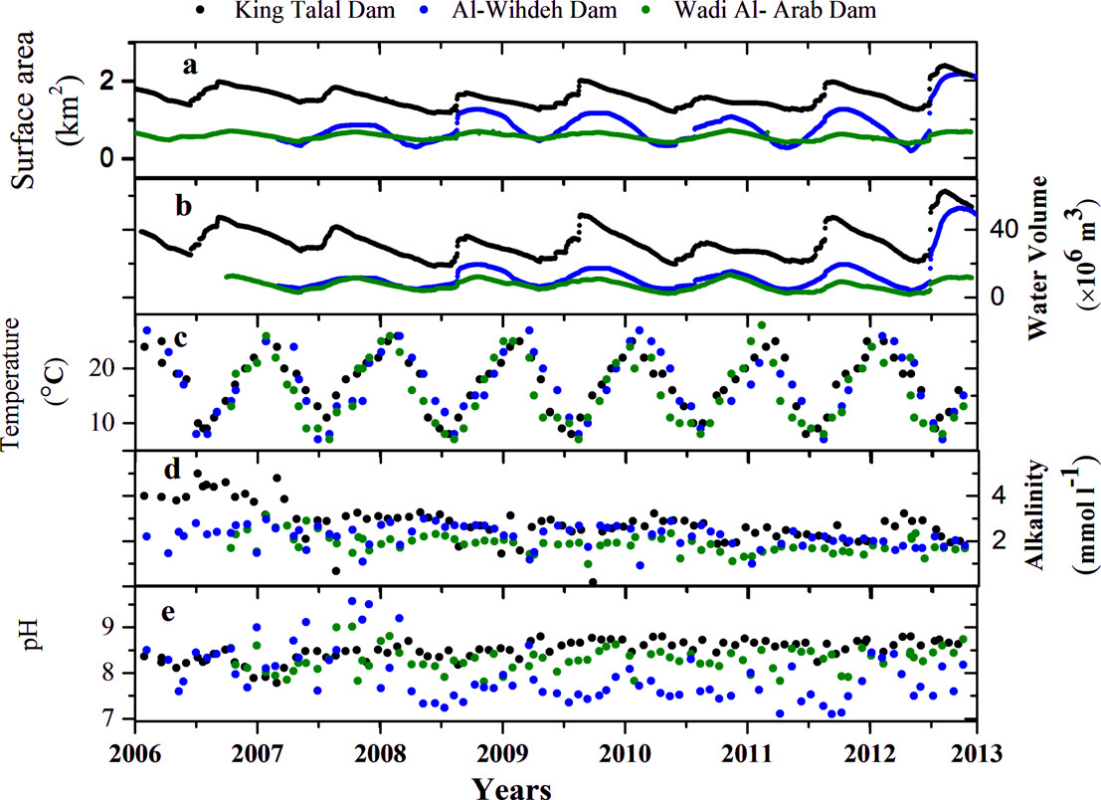
Time series of the physico-chemical parameters in three reservoirs King Talal Dam (black), Wadi Al-Arab (green) and Al-Wihdeh (blue): (a) Daily mean surface area, (b) daily mean water volume, (c) water surface temperature, (d) alkalinity, and (e) pH.

The daily mean of water surface area varied between 1.2 and 2.4 km^2^, 0.37 and 0.76 km^2^, and 0.18 and 2.16 km^2^ in King Talal Dam, Wadi Al-Arab Dam and Al-Wihdeh Dam, respectively (Fig 2A). The surface area varied seasonally due to changes in water volume. Maximum and minimum storage volumes were registered in April and in November, respectively. The storage volume varied also inter-annually with maximum water volumes registered in April 2013 with 13.4 ×10^6^ m^3^ for Wadi Al-Arab Dam, 62.7×10^6^ m^3^ for King Talal Dam and 52.6×10^6^ m^3^ for Al-Wihdeh Dam (Fig 2B). The annual mean water volume stored in the reservoirs was continuously decreasing in King Talal Dam (-13×10^6^ m^3^ y^-1^) and Wadi Al-Arab Dam (-0.9 m^3^×10^6^ y^-1^) and increasing in Al-Wihdeh Dam (0.6×10^6^ m^3^ y^-1^). The available data for King Talal Dam showed that this reservoir received 89% of its inflow from the Az Zarqa River, which is fed by the As Samra Wastewater treatment plant, and from the springs of Amman City. The remaining inflow was from the surrounding tributaries including the flood waters and the discharges of the Az Zarqa desalination plant. The annual mean outflow rate from the reservoirs increased throughout the observation period at King Talal Dam (15330 m^3^ y^-1^) and at Al-Wihdeh Dam (4380 m^3^ y^-1^), while significant changes in water inflow were not observed at both sites. Contrary, the inflow was decreasing (-2190 m^3^ y^-1^) without significant changes of water outflow at Wadi Al-Arab Dam. The resulting water retention times decreased in all three reservoirs (King Talal Dam: -7 d y^-1^, Al-Wihdeh dam: -25 d y^-1^ and Wadi Al-Arab Dam: -9 d y^-1^). The mean water retention times in Al-Wihdeh Dam (247 ±57 d) and Wadi Al-Arab Dam (214 ±57 d) were two times higher than in King Talal Dam (115 ±16 d) (Table 2).

**Table 2:** Summary of the measured and estimated physico-chemical variables and CO_2_ fluxes for the three reservoirs. *n* indicates the number of available water chemistry samples. All numbers are given as mean±SD. The arrows in parentheses indicate significant linear temporal trends (↑ increasing, ↓ decreasing,—not significant for *p*<0.05).

	King Talal Dam	Wadi Al-Arab Dam	Al-Wihdeh Dam
	*n* = 83	*n* = 75	*n* = 78
pH	8.55±0.20 (↑)	8.29±0.26 (-)	7.85±0.0.51(↓)
Alkalinity (mmol l^-1^)	2.85±0.78 (↓)	1.87± 0.4 (↓)	2.28±0.48 (-)
Temperature (°C)	17±5 (-)	15.5±6 (-)	16.4±6 (-)
Salinity (ppt)	1.4±0.3 (↓)	0.8±0.1 (↓)	0.7±0.1 (↓)
Water volume (×10^6^ m^3^)	32±9 (↓)	7±3 (↓)	13±10 (↑)
Retention time (d)	115±16 (↓)	214±57 (↓)	247±57 (↓)
*p*CO_2_ (×10^3^ μatm)	1.0±0.87(↓)	1.1±0.86 (↓)	5.4±4.2 (↑)
Chemical enhancement	8.0±3.1	2.8±1.23	1.6±0.8
*k’*_*600*_ (m d^-1^)	0.62±2.0	0.83±0.42	0.61±0.5
Flux (g CO_2_ m^-2^ d^-1^)	0.7±1.3 (↓)	1±1.6 (↓)	5.3±4.6 (↑)
Total annual emission (×10^5^ g CO_2_ d^-1^)	12±23	5.8± 9.6	45±21

In contrast to Wadi Al-Arab and Al-Wihdeh Dam, we did not observe clear seasonal patterns of the outflow rate at King Talal Dam, although the water inflow rate varied seasonally. Highest and lowest inflow rates for King Talal Dam were registered in March and July-August, respectively. A similar pattern was observed for the inflowing water at Al-Wihdeh Dam. The outflow rate were varied over growing seasons in Wadi Al-Arab Dam and Al-Wihdeh Dam with highest and lowest values in late August and late March, respectively.

Water surface temperature varied seasonally with maximum values of 27°C at the beginning of August and minimum values around 8°C in February (Fig 2C). The mean water temperatures and ranges of variation were of comparable magnitude in all three reservoirs. Salinity in King Talal Dam was nearly twice as high as in the other two reservoirs (Table 2) and decreased significantly in all three reservoirs during the observation period (Al-Wihdeh Dam: -0.005 ppt y^-1^, Wadi Al-Arab: -0.04 ppt y^-1^, King Talal Dam: -0.07 ppt y^-1^). Seasonal variations of salinity were not observed.

Measured alkalinity varied between 0.97 and 3.13 mmol l^-1^ in Wadi Al-Arab Dam, 0.67and 5.0 mmol l^-1^ in King Talal Dam and between 0.92 mmol l^-1^ and 3.0 mmol l^-1^ in Al-Wihdeh Dam (Fig 2D). Exceptionally high alkalinity (>0.4 mmol l^-1^) was measured in 2006 and 2007 in King Talal Dam, which however dropped to values which were comparable to the other two reservoirs in November 2008. After this period, a significant decrease in alkalinity was observed in King Talal Dam (-0.26 mmol l^-1^ y^-1)^ and in Wadi Al-Arab Dam (-0.15 mmol l^-1^ y^-1^). Alkalinity was significantly correlated with salinity in King Talal and Wadi Al-Arab Dams (Table 3).

**Table 3:** Cross-correlation coefficients for the selected parameters: pH, alkalinity (*Alk*) in mmol l^-1^, salinity (*S*) in ppt, temperature (*T*) in °C, *p*CO_2_ in μatm, CO_2_ flux (Flux) in mg CO_2_ m^-2^ d^-1^, water volume (*V*) in m^3^, gas exchange velocity (*k*’_600_) in m d^-1^ and chemical enhancement factor (*α*). For each combination of parameters, the three numbers are the cross-correlation coefficients observed for King Talal (upper), Wadi Al-Arab (middle) and Al-Wihdeh (lowest) dams. The most significant correlation coefficients (*p*<0.05) are marked bold, while *p*<0.1 for the remaining correlations. Not significant correlations are marked as x.

	pH	*Alk*	*S*	*T*	*p*CO_2_	Flux	*V*	α
*Alk*	**-0.5**		**0.47**	x	**0.77**	**0.77**	0.32	x
	**-0.28**		**0.6**	x	**0.5**	**0.35**	x	x
	**-0.23**		x	x	**0.32**	**0.4**	-0.23	-0.31
*S*	-0.3	**0.47**		x	**0.4**	0.4	x	x
	-0.35	**0.6**		x	**0.5**	x	x	x
	x	x		x	x	x	x	x
*T*	x	x	x		x	x	0.3	**-0.68**
	x	x	x		x	0.38	x	**0.88**
	0.31	x	x		-0.22	x	x	**0.4**
*p*CO_2_	**-0.89**	**0.77**	**0.4**	x		**0.98**	0.21	x
	**-0.95**	**0.5**	**0.5**	x		**0.83**	x	x
	**-0.95**	**0.32**	x	-0.22		**0.91**	x	x
Flux	**-0.86**	**0.77**	0.4	x	**0.98**		0.26	x
	**-0.78**	**0.35**	x	0.38	**0.83**		x	x
	**-0.85**	**0.4**	x	x	**0.91**		x	-0.29
*V*	x	0.32	x	0.3	0.21	0.26		x
	x	x	x	x	x	x		x
	x	-0.23	x	x	x	x		x
α	x	x	x	**-0.68**	x	x	x	
	x	x	x	**0.88**	x	x	x	
	x	-0.31	x	**0.4**	x	-0.29	x	
*k*’_600_	x	x	-0.31	0.36	x	x	x	**-0.84**
	x	x	x	x	x	0.28	x	x
	0.28	x	x	x	-0.25	x	x	**-0.83**

The pH values in the three reservoirs varied between 7.10 and 9.81 (Fig 2E). The mean pH in King Talal Dam (8.50) was consistently higher than the mean pH in Al-Wihdeh (7.85) and Wadi Al-Arab Dam (8.29) (Table 2). Exceptionally high pH values (pH>9) were observed during the initial filling of Al-Wihdeh Dam in 2007 and 2008. In the years following the initial filling, the pH in Al-Wihdeh Dam was significantly lower than in the other two reservoirs. The pH increased during the observation period in King Talal Dam (0.07 y^-1^) and decreased in Al-Wihdeh Dam (-0.1 y^-1^).

### 3.2 CO_2_ partial pressure

**Fig 3:**
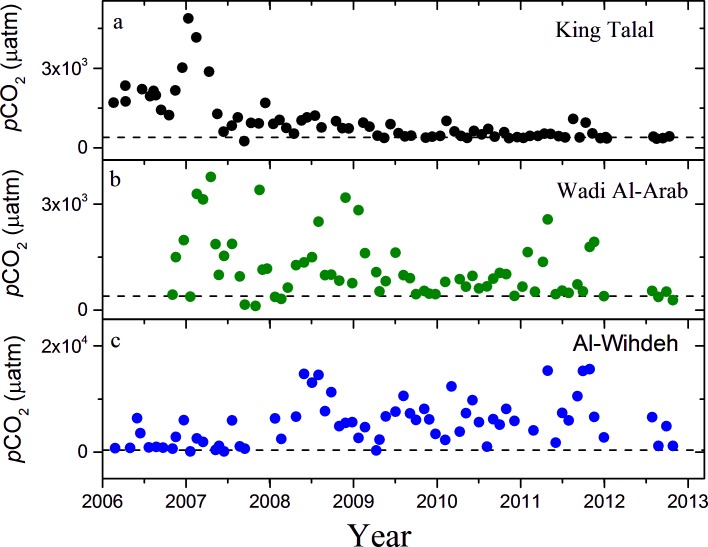
Time series of the monthly estimates of *p*CO_2_ for (a) King Talal Dam (black), (b) Wadi Al-Arab Dam (green), and (c) Al-Wihdeh Dam (blue). The dashed lines show the mean atmospheric *p*CO_2_ (396 μatm).

The three reservoirs were supersaturated with CO_2_ with respect to the atmosphere for most of the time. 88% of the water samples from Wadi Al-Arab and Al-Wihdeh Dam, and 82% of the water samples from King Talal Dam had CO_2_ partial pressures, which were exceeding the mean atmospheric value. The *p*CO_2_ values in King Talal Dam ranged between 250 and 4900 μatm, in Wadi Al-Arab Dam between 121 and 3776 μatm, and in Al-Wihdeh Dam between 98 and 15616 μatm (Fig 3). The mean *p*CO_2_ and its standard deviation was about five times higher in Al-Wihdeh Dam (5400±4200 μatm) than in the other two reservoirs, which had comparable mean CO_2_ partial pressures of 1100 μatm (Table 2). While a clear seasonal pattern of *p*CO_2_ could not be observed, it was continuously decreasing with time in King Talal Dam (-312 μatm y^-1^) and in Wadi Al-Arab Dam (-186 μatm y^-1^), while it was increasing at Al-Wihdeh Dam (879 μatm y^-1^). The increasing trend in Al-Wihdeh Dam, however, was mainly caused by low CO_2_ partial pressure during the initial filling period of the reservoirs until 2009. The *p*CO_2_ in Al-Wihdeh Dam remained rather constant at high levels after the year 2010. *p*CO_2_ was significantly correlated with pH and alkalinity in all three reservoirs and it was additionally correlated with salinity in King Talal and Wadi Al-Arab Dam (Table 3).

### 3.3 Fluxes

**Fig 4:**
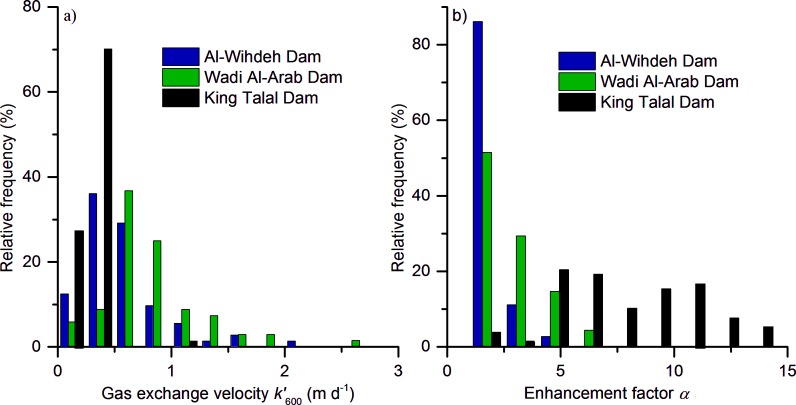
The frequency distribution of (a) gas exchange velocity and (b) chemical enhancement factor for the three reservoirs.

The wind-speed dependent gas exchange velocity *k’*_600_ was generally lower than 1 m d^-1^, with 94%, 86% and 78% of the values in King Talal, Al-Wihdeh and Wadi Al-Arab Dam, respectively (Fig 4A). The mean gas exchange velocity in Wadi Al-Arab Dam (0.83±0.42 m d^-1^) was slightly higher than those in King Talal (0.62±2.2 m d^-1^) and Al-Wihdeh (0.61±0.5 m d^-1^) Dam (Table 2).

Chemical enhancement was an important process for CO_2_ evasion in all three reservoirs and lead to an up to fourteen fold increase of the gas exchange. The enhancement factors ranged between 1.4 and 6.9 in Wadi Al-Arab Dam, 1.0 and 14.4 in King Talal Dam and between 1.3 and 4.7 in Al-Wihdeh Dam (Fig 4B) and the mean enhancement factor was highest at King Talal Dam (Table 2). Chemical enhancement was significantly correlated with water temperature, although the sign of the correlation coefficients differed among the three reservoirs (Table 3).

**Fig 5:**
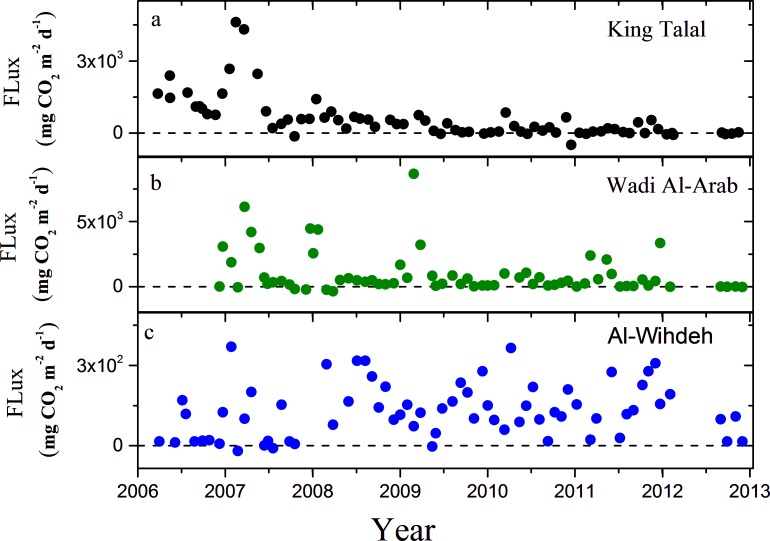
Time series of the CO_2_ flux for the three reservoirs in (a) King Talal Dam (black), (b) Wadi Al-Arab Dam (green), and (c) Al-Wihdeh Dam (blue). The y-axes is in different scale and the dashed lines were fixed at zero.

**Fig 6:**
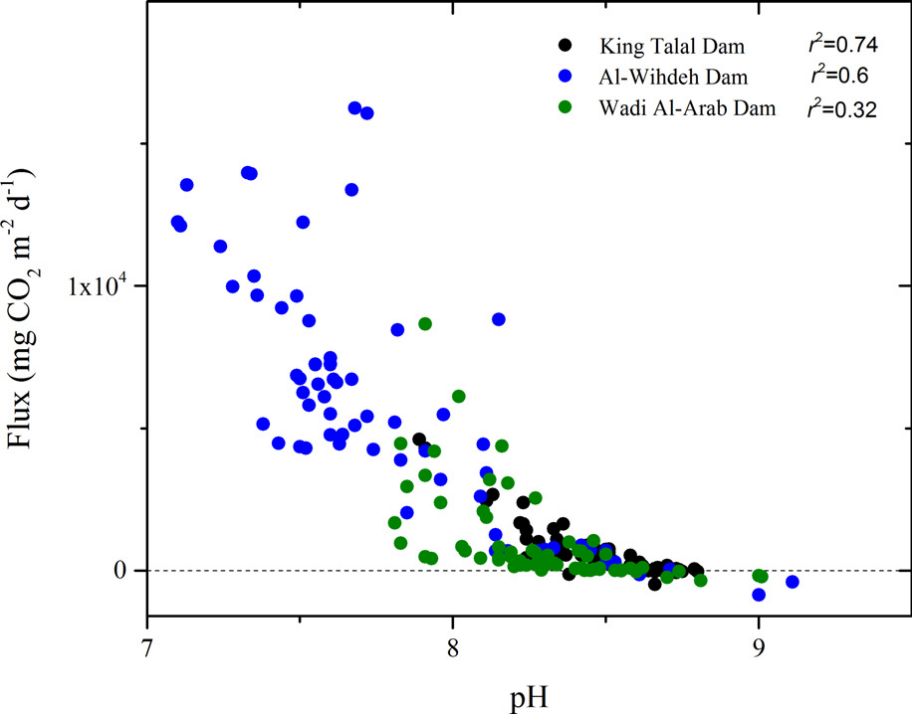
Atmospheric fluxes of CO_2_ as a function of pH in the three reservoirs. R2 denote to the linear regression.

The CO_2_ fluxes varied widely among the reservoirs and among sampling dates but except for the year 2013, where the available data did not cover the full year, annual mean fluxes were positive, i.e. the reservoirs were a net source for atmospheric CO_2_ (Fig 5). Persistent seasonal patterns in the monthly flux estimates were not observed. The highest mean flux and range of variation were registered in Al-Wihdeh Dam with values between -1157 and 17440 mg CO_2_ m^-2^d^-1^, where negative fluxes were mainly observed during the initial filling of the reservoir in 2007 and 2008. The estimated fluxes ranged between 448 and 8538 mg CO_2_ m^-2^ d^-1^ in King Talal Dam and between -348 to 8657 mg CO_2_ m^-2^ d^-1^ in Wadi AL-Arab Dam. The annual mean flux of CO_2_ decreased significantly throughout the observation period in King Talal Dam (-686.5 mg CO_2_ m^-2^d^-1^ y^-1^) and Wadi Al-Arab Dam (-264 mg CO_2_ m^-2^d^-1^ y^-1^). The fluxes were significantly correlated with pH, alkalinity and *p*CO_2_ in all three reservoirs (Table 3), although pH can be considered as the most sensitive input parameter in our calculations (Fig 6). The resulting total emission rates were highest in Al-Wihdeh Dam (Table 2), which had the highest *p*CO_2_.

### 3.4 Sensitivity analysis

To test the relative contributions of temporal variations of the different input data on the estimated mean values of CO_2_ partial pressure and flux, we compared our results to corresponding estimates for which one variable was kept constant at its mean value. This sensitivity analysis indicates that variations in alkalinity, salinity and temperature had only minor effects on mean values of *p*CO_2_ and fluxes (< 5%). Using a constant mean pH value for each reservoir resulted in an increase of estimated *p*CO_2_ of up to 10% for Al-Wihdeh and Wadi Al-Arab Dams, and 15% for King Talal Dam. The pH affects not only the carbon equilibrium calculations but also the chemical enhancement factor. Thus, the sensitivity of the flux to variations in pH can be expected to be high. Whereas, excluding the measured ion concentrations in the chemical equilibrium calculations resulted in an overestimation of the mean *p*CO_2_ by 10% in Al-Wihdeh, and 15% in King Talal and Wadi Al-Arab Dam and corresponding increases of CO_2_ fluxes.

The CO_2_ fluxes were most sensitive to wind speed, which is affecting both the enhancement factor and the gas exchange velocity. Using a constant mean wind speed resulted in an increase of the mean flux by a factor of 3.4, 2.5 and 2.6 for King Talal, Al-Wihdeh and Wadi Al-Arab Dams, respectively.

## Discussion

### 4.1 Uncertainties and chemical enhancement of the CO_2_ flux

The flux estimates discussed above are subject to uncertainties caused by uncertainties of the measured input data, as well as by uncertainties arising from the limited spatial and temporal resolution of these data. The sensitivity analysis revealed that the nonlinear dependence of the estimated *p*CO_2_ on alkalinity, pH, salinity, temperature and ion concentration caused only moderate uncertainties of the estimated fluxes of up to 10 to 15%. In accordance with findings of Abril *et*. *al*. [30], these uncertainties cause an overestimation of the fluxes. An additional overestimation of comparable magnitude can occur in organic-rich and acidic waters, where organic acids make a significant contribution to the total alkalinity [30]. However, there is no available data that can address the possible contribution of organic acids to the total alkalinity.

**Fig 7:**
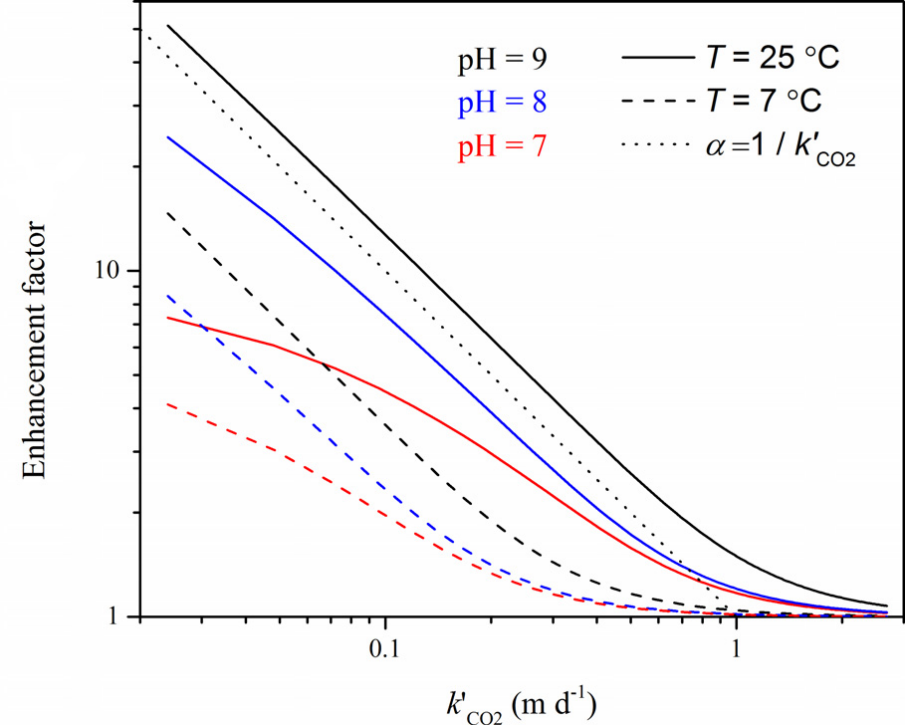
Chemical enhancement factor as a function of unenhanced gas exchange velocity *k’*_CO2_ for three different pH values (color) and at two different temperatures (solid line: 25°C, dashed line: 7°C). The dotted line indicates a reciprocal relationship between both parameters and therewith a resulting exchange coefficient, which is independent of wind speed. Chemical enhancement further depends on salinity, which was fixed at a value of 0.8 ppt.

Much higher are the uncertainties of the flux estimates related to wind speed. While the gas exchange velocity was assumed to be linearly related to wind speed, its non-linear effect on the chemical enhancement factor can cause several-fold underestimations of the fluxes. The CO_2_ exchange with the atmosphere for the three reservoirs was enhanced by up to fourteen fold, with an average enhancement factor of four. This value is higher than that considered for natural lakes, where it was assumed to be three [31]. Chemical enhancement was not considered in the most recent estimation of global CO_2_ emissions from inland waters [4]. The importance of chemical enhancement has been addresses in lab experiments and in various aquatic ecosystems [24, 31, 32]. The enhancement factor increases for increasing pH and temperature and is particularly high at low wind speeds, when the exchange velocity is low [32]. The relationship between the wind-speed dependent gas exchange velocity *k’*_600_ and the chemical enhancement factor is shown for a range of observed pH and temperatures in Fig 7. For pH≥8, the enhancement factor tends to become reciprocally related to *k’*_600_, at small exchange velocities. This dependence results in a constant, i.e. wind-speed independent, enhanced exchange velocity (*k’*_600_ ∙*α*, cf. Eq 1). Chemical enhancement thus provides a mechanism, which is capable to off-sets the decrease of the gas exchange velocity for decreasing wind speed.

### 4.2 Variability of *p*CO_2_ and fluxes

The observed seasonal patterns of water volume at the three reservoirs were mainly caused by the balance between the outflow and inflow rates. In contrary to King Talal Dam, the seasonal pattern of the water volume at Wadi Al-Arab Dam resulted from the seasonality of the inflowing water. On the other hand, decreasing outflow and increasing inflow rates at Al-Wihdeh Dam during March, and the opposite during July-August, caused the seasonal pattern of the water volume in Al-Wihdeh Dam. However, the minimum and maximum values of inflow and outflow rates may refer mainly to the seasonality of precipitation and consumption rates, respectively.

The high salinity at King Talal Dam can be related to the water quality of the inflowing water, i.e. to the high amount of treated wastewater from the As Samra Wastewater treatment plant. Further studies should take into account the processes, which regulate the variability of the physico-chemical characteristics of the reservoirs. The significant correlation between salinity and alkalinity in King Talal Dam may explain the continuous decrease of the annual mean alkalinity during the observation period. The relationship between water salinity and alkalinity can be linked to the removal of freshwater, i.e. the decreasing contribution of spring waters, which is reflected by salinity changes [33–35]. The long term decrease of the annual mean *p*CO_2_ and CO_2_ flux that was observed in Wadi Al-Arab Dam and at King Talal Dam was mainly caused by decreasing alkalinity. Mean gas exchange velocity in King Talal dam was lower than in the other reservoirs (Table 2), which resulted in the highest enhancement factor in King Talal Dam (Fig 4B). The negative relationship between the chemical enhancement factor and the gas exchange velocity (Fig 7) was proposed by the model of Hoover and Berkshire [27]. Chemical enhancement varied strongly among the three reservoirs under study, indicating that using the fixed default value which was proposed by Cole *et*. *al*. [36] can result in a high degree of uncertainty of the flux estimates.

The observed change in surface area for the three reservoirs over the growing seasons resulted a substantial change in the total emission. Therefore, the variability in the hydrological characteristics for the three reservoirs, which was causing the change in surface area, is strongly linked to the water management practice, i.e. the balance between inflow and outflow. The emerged sediments in the seasonally varying dry belts can be additional, potentially strong sources of CO_2_ emissions, which were not included in our estimates. von Schiller *et*. *al*. [37] found extraordinary high areal emission rates from Mediterranean rivers during temporary dry periods, which were exceeding the emission rates of both water surfaces and soil. Future studies, which aim at a complete greenhouse gas budget of reservoirs should take these emissions into account.

### 4.3 CO_2_ fluxes in a global context

We estimated the atmospheric emission rates of CO_2_ from three reservoirs located in the lower Jordan watershed using water quality monitoring data. The same methodological approach has been applied in the past to obtain regional to global scale estimates of CO_2_ fluxes from reservoirs and other inland waters [4, 11, 38]. The total mean CO_2_ flux from the three reservoirs of 3.3 g CO_2_ m^-2^ d^-1^ is in the range of fluxes reported for tropical and subtropical reservoirs in global assessments and corresponds to about 3-fold of the most recent mean flux from global lakes and reservoirs (Table 4). Moreover, the total mean CO_2_ flux from the reservoirs exceeded those reported for reservoirs located in other semiarid regions [12, 13].

**Table 4:** Comparison of the estimated mean flux of CO_2_ with zonal and global estimates from the recent literature.

	CO_2_ flux	Number	Reference
	(g CO_2_ m^-2^ d^-1^)	of reservoirs	
**Global hydroelectric reservoirs**	**1.5**		[7]
Temperate zone	0.4	85	
Tropical zone	3.1		
Younger than 20 years	3.7		
Older than 20 years	1.2		
**Global reservoirs**	**1.87**	**149**	[9]
Subtropical reservoirs	0.78	36	
Tropical reservoirs	4.0	20	
**Global reservoirs**	**1.8**	**22**	[8]
Temperate zone	1.4	17	
Tropical zone	3.5	5	
**Global lakes and reservoirs**	1.0	7939	[4]
**This study** (subtropical reservoirs)	3.3	3	Table 2 (area-weighted average)

The fluxes differed among the three reservoirs and while the mean values of the more than 20 year old reservoirs King Talal Dam and Wadi Al-Arab Dam are comparable to the global-mean estimate for lentic ecosystems of 1 g CO_2_ m^-2^ d^-1^ [4], the emission rate from the newly established Al-Wihdeh Dam exceeds this value more than 5-fold. This relatively high emission rate is in the range of fluxes measured in other newly established reservoirs, both in the tropical as well as in the boreal zone [39] but is about 40% higher than the global mean flux from reservoirs younger than 20 years estimated by Barros *et*. *al*. [7]. In contrast to these studies, however, a significant decrease of the CO_2_ flux during the first years of operation was not observed in Al-Wihdeh Dam. Previous studies indicated that the main source of carbon in the young reservoirs are originated flooded biomass [8, 40], which was decomposed in the first three years [8]. However, continuous increasing in the water volume in Al-Wihdeh dam indicates that the reservoir did not reach the first year of full filling and the biomass is still flooding. The fluxes from the two older dams decreased significantly during the observation period and with annual mean *p*CO_2_ approaching atmospheric equilibrium the reservoirs appear to be a relatively weak source for atmospheric CO_2_ after about 25 years of operation in comparison to other inland waters [4]. While the declining fluxes with increasing reservoir age can potentially be caused by the declining availability of degradable carbon in the flooded biomass and soil organic matter [7], the simultaneously observed decreasing trends in water retention time and salinity suggest that also changes of water quality and water management contribute to the observed trends in CO_2_ fluxes from King Talal and Wadi Al-Arab Dam.

While previous studies have mainly focused on hydropower reservoirs, which are typically located in regions where water resources are more abundant [7, 39, 41], the irrigation and drinking water reservoirs studied here are subject to strong anthropogenic pressure on declining water resources. The fact that the studied reservoirs receive large fractions of their inflowing water as treated waste water suggests that besides reservoir age, also eutrophication and primary production can have strong effects on their carbon budgets. Primary production was measured in Wadi Al-Arab Dam in 2001 by Saadoun *et*. *al*. [42] to be 92 mg C m^-3^ h^-1^. This rate corresponds to an areal CO_2_ uptake rate of ≈8 g CO_2_ m^-2^ d^-1^ only from production within the first meter of the water column, and therewith exceeds the estimated atmospheric emission rates. Changing nutrient concentration and water retention time, which affect the rate of primary production in reservoirs [42], can therefore be expected to also affect the amount of carbon emitted to the atmosphere. However, the high rates of the primary production in comparison to the observed CO_2_ flux suggest a strong diurnal variation of *p*CO_2_ and CO_2_ flux, which can result in a substantial bias in the annual estimates of *p*CO_2_ and exchange rate because the monthly sampling was restricted to day time. Diurnal variation of *p*CO_2_ and exchange rate has been observed in various aquatic ecosystems [12, 43], which indicates the importance of high temporal resolution measurements for further studies.

The inorganic carbon, which is recycled within the reservoir by primary production can partly be stored in the sediments [44] or it can be emitted during and after water usage either from soils after irrigation or during water treatment for drinking water production. A more complete analysis of the carbon and greenhouse gas budget of the reservoirs would therefore require the availability of data on organic and inorganic carbon burial within and export from the reservoirs [9]. Data on sedimentation rates in the reservoirs are available for King-Talal Dam, where the mean annual sediment yield was estimated to be 0.63 10^6^ m^3^ [18]. This sediment yield corresponds to a mean increase of sediment thickness of 0.3 m y^-1^. Such high sedimentation rates can be expected to lead to high production and emission rates of methane [45]. Therefore, further studies are needed to quantify and identify the total C released from these systems. The actual production rate of methane, however, depends strongly on the quantity and quality of organic carbon in the sediments, as well as on the physical characteristics. Predictors for methane emission rates, which are based on bulk water and sediment quality data require data for dissolved methane concentration in water [46], which are not readily available as the data required to estimate CO_2_ evasion.

## Conclusions

This study is providing estimates of CO_2_ fluxes from reservoirs located in a semi-arid region from which a limited number of studies were addressing the magnitude CO_2_ evasion rates. The magnitude of observed fluxes is most comparable to those from tropical reservoirs [9, 12, 13] and generally agrees with estimates from larger-scale assessments. Moreover, our analysis revealed two important points: At first, the relatively high fluxes were, to a large extent, controlled by chemical enhancement of gas transfer. Chemical enhancement becomes important at high pH and low wind speed, where this process is capable to off-set the linear dependence of the gas exchange velocity on wind speed. Nevertheless, the majority of recently published regional to global-scale flux estimates did not take chemical enhancement into account and potentially underestimate CO_2_ emission rates from inland waters. Second, our analysis revealed pronounced temporal variations of the fluxes, which can be related to changes in water and reservoir management. While recent research has mainly been focusing on constraining regional and global scale flux estimates and carbon budgets, our results indicate an urgent need also for assessing temporal trends of the fluxes caused by anthropogenic activities. Potential effects of human activities on CO_2_ emissions from inland waters has been reviewed by [3, 5], but mainly consider the construction of reservoirs and not water and reservoir management practices that also can affect the total emission.

## Supporting Information

S1 Dataset Physico-chemical and hydrological parameters of the three studied reservoirs. (XLSX)
